# Biological Characteristics and Energy Metabolism of Migrating Insects

**DOI:** 10.3390/metabo13030439

**Published:** 2023-03-17

**Authors:** Xiaokang Li, Yan Zhou, Kongming Wu

**Affiliations:** 1 College of Plant Protection, Shenyang Agricultural University, Shenyang 110866, China; 2 Guangdong Laboratory for Lingnan Modern Agriculture, Guangzhou 510640, China; 3 State Key Laboratory for Biology of Plant Diseases and Insect Pests, Institute of Plant Protection, Chinese Academy of Agricultural Sciences, Beijing 100193, China

**Keywords:** insect migration, energy substances, flight and reproduction, juvenile hormone

## Abstract

Through long-distance migration, insects not only find suitable breeding locations and increase the survival space and opportunities for the population but also facilitate large-scale material, energy, and information flow between regions, which is important in maintaining the stability of agricultural ecosystems and wider natural ecosystems. In this study, we summarize the changes in biological characteristics such as morphology, ovarian development, reproduction, and flight capability during the seasonal migration of the insect. In consideration of global research work, the interaction between flight and reproduction, the influence and regulation of the insulin-like and juvenile hormone on the flight and reproductive activities of migrating insects, and the types of energy substances, metabolic processes, and hormone regulation processes during insect flight are elaborated. This systematic review of the latest advances in the studies on insect migration biology and energy metabolism will help readers to better understand the biological behavior and regulation mechanism of the energy metabolism of insect migration.

## 1. Introduction

The long-distance migration of animals is a universal ecological phenomenon in periodically changing environmental conditions and specific life cycles [1]. Insects are the most abundant and widely distributed animals in the world [2], and many insects (Lepidoptera, Orthoptera, Hemiptera, Odonata, etc.) have migratory habits. Migratory insects can regularly migrate from one habitat to another in groups in certain seasons and then complete life activities such as feeding, mating, and oviposition by using variable spatiotemporal resources, reflecting their adaptation to the environment [3,4,5]. The occurrence of migration is simultaneously affected by external environmental factors and the insects’ own behavior and physiological state (species, individuals, etc.) [3,6]. Many factors, such as the photoperiod changes, poor nutrition, inappropriate temperature changes, increased population density, and increased numbers of natural enemies or pathogens, can cause habitat deterioration, which can induce migration [6,7,8]. The occurrence of insect migration requires stimulation by external environmental conditions, but only when these factors act on a specific period of insect development can they regulate and affect migration behavior [8,9].

The most important reason for insect migration is the demand for reproduction. Flight and reproduction are high-energy-consuming processes. The energy substances required for insect flight are basically the same as those required for reproductive activities. In the case of the limited storage of energy substances, energy allocation will affect the trade-off between migration and reproduction [10]. Energy substances distribution and hormone regulation in insects play crucial roles in both migratory flight and reproduction. The synthesis and metabolism of energy substances in insects are regulated by hormones, such as the adipokinetic hormone (AKH), insulin, the juvenile hormone (JH), and others [11,12,13,14,15]. As important endocrine hormones, the juvenile hormone and insulin directly regulate the growth, development, metamorphosis, reproduction, and other physiological processes of insects [16,17,18], and the level of the juvenile hormone is closely related to insect migration [19,20,21]. The insulin signaling pathway can regulate the metabolism of carbohydrates, lipids, and proteins in insects [22] and is also involved in the regulation of wing type differentiation among migratory insects [18,23].

Based on the long-term monitoring of migratory insects, we summarized and discussed the seasonal variations in biological characteristics such as morphological changes, ovarian development, reproduction, and flight capability in the process of insect migration. The interaction between reproduction and flight and the metabolism and regulation of energy substances during insect migration were reviewed in order to better understand the physiological basis and regulation mechanism of insect migration.

## 2. Changes in Biological Characteristics during Insect Migration

### 2.1. Variations in Morphological Parameters

High-altitude migratory insect populations across the Bohai Sea were monitored from April to October in recent decades using searchlight traps and a scanning entomological radar on Behuangcheng Island (BHC) in the center of the Bohai Strait. The wing load and flight muscle ratio of obligate migratory insects, such as the black cutworm *Agrotis ipsilon* (Lepidoptera: Noctuidae) and the common cutworm *Agrotis segetum* (Lepidoptera: Noctuidae), are significantly higher than those of facultative migratory insects, such as the cotton bollworm *Helicoverpa armigera* (Lepidoptera: Noctuidae), and the body shape and wing type of the obligate migratory insects are slenderer, which may be one of the morphological factors that give them a stronger flight capability than facultative migratory insects [24]. The long-term monitoring of migratory insects on BHC found that northward migrants in spring and summer had larger body sizes than southward migrants. For example, the body length and weight of *H. armigera* migrating north from the North China Plain were 1.2- and 1.33-fold larger, respectively, than those migrating south from the Northeast Plain, respectively [25]. Similar morphological differences were also found in migratory populations of the beet armyworm, *Spodoptera exigua* (Lepidoptera: Noctuidae), and *Athetis lepigone* (Lepidoptera: Noctuidae) (unpublished data), which may be related to the differences in the host plants and photoperiods between the two plains.

The wing morphology of migratory insects varies among different populations and is prone to damage during flight. Long-distance migratory populations of monarch butterflies, *Danaus plexippus* (Lepidoptera: Nymphalidae), have larger bodies and wing loading than nonmigratory populations [26]. The painted lady butterfly, *Vanessa cardui* (Lepidoptera: Nymphalidae), suffers varying degrees of wing damage after long-distance flights, which may cause negative effects on many biological processes, such as flight capacity, body color, body temperature regulation, etc. [27].

### 2.2. Changes in Flight Behavior

The wingbeat frequency of oversea migrants varies at the order, family, and species levels, which is related to the different body structures of insects [28,29]. Laboratory measurement results showed that the wingbeat frequency of Lepidoptera was 6.71–81.28 Hz, that of Neuroptera was 19.17–30.53 Hz, and that of Odonata was 18.35–38.01 Hz [29]. While Lepidoptera have a wide range of wingbeat frequencies, the wingbeat frequency of Noctuidae pests is mainly concentrated at 35–45 Hz, and temperature is the main environmental factor affecting it. *H. armigera* flapped had the highest wingbeat frequency at 28–32 °C, while the oriental armyworm, *Mythimna separata* (Lepidoptera: Noctuidae), and *A. ipsilon* had the highest wingbeat frequency at 28 °C and 24 °C, respectively [30].

Insects show obvious common orientation behavior in the process of cross-sea migration, and the orientation has seasonal adaptability. *H. armigera* migratory populations orient toward the northeast in spring but have no clear flight orientation in summer [31]. In autumn, *M. separata* orient toward the southwest [32], and migratory insects such as the beet webworm, *Loxostege sticticalis* (Lepidoptera: Noctuidae), *A. ipsilon*, and *S. exigua* exhibit the same behavior [33]. Most migratory insects traveling across the Bohai Strait usually fly at an altitude of less than 1500 m above sea level. Migrating insect populations constantly adjust their flight altitude to select the most suitable wind direction, wind speed, and temperature to gather into layers at the most appropriate height [34,35,36,37]. During migration, *H. armigera* usually fly at altitudes of 200–500 m [37], and the flight distance can reach 150–300 km in a single night. In early summer, *H. armigera* congregates in the layers with temperature inversion and the highest wind speed [34]; in mid-summer, its stratification height is related to the maximum wind speed and wind shear but not temperature [36]. In autumn, *M. separata* gathers in the layer 50–500 m above sea level and migrates southward [32]. The migration altitude of the globe skimmer dragonfly, *Pantala flavescens* (Odonata: Libellulidae), is generally lower than 1000 m at sea level, and its stratification height is consistent with that of the temperature inversion layer [38].

### 2.3. Feeding Behavior during Migratory Flight

Insects will feed on plant nectar to supplement their nutrition and meet their energy requirements during long-distance migration [39]. Affected by physiological needs and the differences in nutrient composition, migratory insects have a preference for nectar source plants. It was found that the migratory hoverfly *Episyrphus balteatus* (Diptera: Syrphidae) population visited a minimum 1012 flowering plant species (429 genera, 91 families, 39 orders), among which Compositae, Gramineae, and umbelliferous and leguminous plants accounted for the highest proportion, and herbaceous plants accounted for a higher proportion than woody plants [40]. During cross-sea migration, the clover cutworm, *Scotogramma trifolii* (Lepidoptera: Noctuidae), fed on at least 42 families and 92 species of nectar plants, and a high proportion of migrants carried pollen from Compositae, Amaranthaceae, and Pinaceae plants [41]. *H. armigera* fed on at least 32 species of nectar plants in 28 families, carrying the highest amount of pollen from Oenothera plants [42]. *M. separata* fed on 13 species of plants belonging to at least 9 families, mainly in the dicotyledonous class of the angiosperm phylum [43]. Similar to *M. separata*, Agrotis pests also prefer to feed on nectar plants of the dicotyledonous class of angiosperms. During long-distance migration, *A. ipsilon* visited more than 33 species of plants and *A. segetum* fed on more than 40 species of plants; *A. segetum* preferred herbaceous plants [44], while *A. ipsilon* preferred woody plants [45]. Affected by phenological changes, the pollen-carrying rates of these migratory insects have showed seasonal differences, with a higher rate for the northward migrating population than for the southward migrating and mixed migrating populations [42,43,44,45].

Indoor simulation experiments found that poor nutritional and environmental conditions during the adult stage could stimulate the migration of *H. armigera* [46], and feeding on honey water could significantly enhance the flight and reproductive ability. For example, the number of eggs laid by a single migrating *H. armigera* female after feeding on 10% honey water reached 839, which was 2.82 times that of individuals feeding on water [42]. Therefore, honey water supplementation can improve the ecological adaptability of migrating populations. However, not all plant nectar is beneficial to insects, and the suitability of nectar for different insects varies. For example, pine pollen can significantly improve the fecundity of *A. lepigone* but significantly inhibit the fecundity of *M. separata*, the loreyi leafworm, *Leucania loreyi* (Lepidoptera: Noctuidae), and *S. exigua* [47].

### 2.4. Changes in Reproductive Status and Capacity

**Figure 1:**
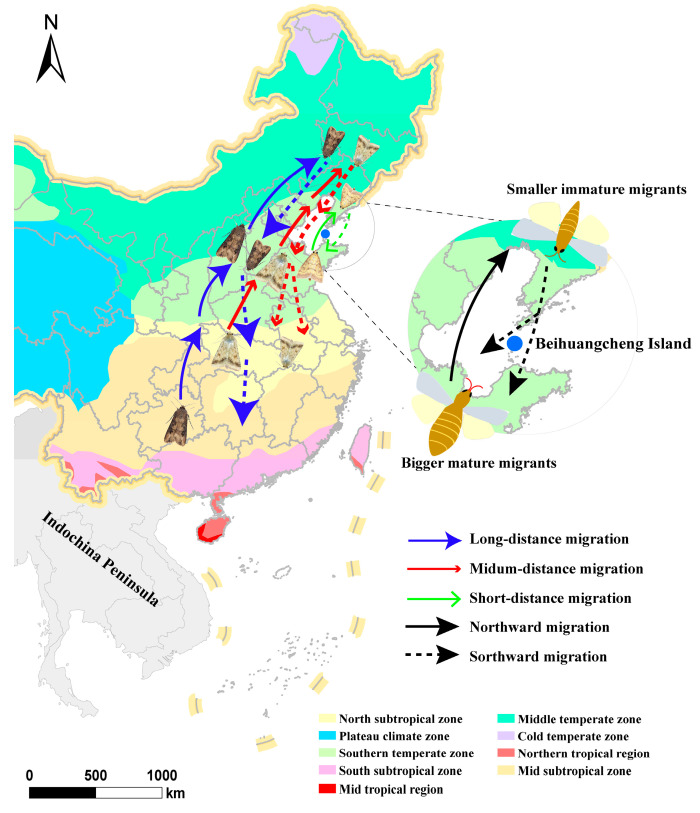
Schematic diagram of migration patterns and physiological characteristics of insects across Bohai Sea.

The anatomical results showed that the ovarian development level, mating proportion, and mating frequency of individual cross-Bohai Sea migratory insects showed a decreasing trend over time [48,49,50,51,52,53]. The proportion of sexually mature and mated female moths of the northward migration population in spring was significantly higher than that of sexually immature and virgin female moths, while the reproductive pattern was the opposite of that of the southward migration population in autumn [48,49,50,51,52,53]. This phenomenon showed that the insect populations that migrated to the Bohai Sea in spring had already completed mating activities during migration, and migrants entered the spawning and breeding stage after landing at the destination. The individual migratory insects of the southward population captured in autumn were in the immature stage. According to the overwintering regions and characteristics of cross-Bohai-Sea migratory insect populations, their migratory activities can be divided into short-distance migration (<200 km), medium-distance migration (200–500 km), and long-distance migration (>500 km) [54]. Integrating the geographical location of BHC and the temporal distribution of captured migratory insects, these findings suggest that the migration behavior of insects starts from the immature ovary stage. The reproductive capacity of the northward population in spring was found to be significantly higher than that of the southward population in autumn. The spawning capacity of *H. armigera* in spring was 8.8 times that of those in autumn in the absence of supplementary nutrition. Nutritional supplementation can significantly improve the reproductive capacity of migrating *H. armigera*, and the increasing effect is most significant for insects in the early stage of growth and development [42]. Insect migration patterns across the Bohai Sea are shown in Figure 1.

## 3. Regulatory Mechanism of Insect Migration and Reproduction Interaction

### 3.1. Relationship between Insect Migration and Reproduction

The interaction between migration and reproduction is the main factor that determines the population dynamics of migratory insects. According to the classic “oogenesis-flight syndrome” [55,56,57], migratory insects begin migration at the juvenile stage; before migration, ovarian development temporarily stops, and reproductive activities such as ovum development and oviposition are completed after long-distance flight. This shows that insects have a strong flight capability before reproduction. The reproductive progress will gradually reduce migratory insects’ flight capability. The flight capability of *M. separata* was found to decrease significantly with increased spawning quantity and duration [58]. After mating, the flight distance and speed of *A. segetum* were significantly lower than those of unmated individuals [59]. The flight speed of the cabbage moth, *Mamestra brassicae* (Lepidoptera: Noctuidae), after mating and reproduction was gradually slower than that of unmated individuals [60]. Therefore, comparing the variation trend of flight capability before and after adult reproduction can determine whether the insect population exhibits oogenesis-flight syndrome.

The flight of migratory insects consumes energy storage substances, which imposes a reproductive cost, such as prolonging pre-oviposition, decreasing oviposition, and reducing lifespan [61,62]. The flight of 1-day-old *S. exigua* led to a prolonged period of adult oviposition, a reduced quantity of eggs, decreased mating times, and a shortened lifespan of female adults [63]. The first flight of *L. sticticalis* at 1 day after eclosion resulted in increased pre-oviposition and delayed oviposition [64]. The lifespan, the reproductive duration, and the number of offspring of the soybean aphid, *Aphis glycines* (Hemiptera: Aphididae), decreased with the increase in flight distance [65]. The anterior and posterior wings and indirect flight muscles of the long-winged brown planthopper, *Nilaparvata lugens* (Hemiptera: Delphacidae), are fully developed, and the adult individuals are capable of long-distance flight [66]. Short-winged brown planthoppers cannot fly, but their reproductive ability is stronger than that of long-winged brown planthoppers [67,68].

Migration may not lead to the decreased reproductive fitness of insects. Affected by the actual physiological conditions during flight, the reproductive ability of some insect migrants may not be lower than that of residents; migration flight has no significant effect on the reproductive ability of insects. Reproductive system development in some migratory insects even depends on the occurrence of migratory flight, which can promote the rapid development of the reproductive system and the initiation of reproductive activities. The flight of *S. exigua* adults was not found to significantly affect their reproductive ability, except for adults 1 day post-emergence [63]. The flight of 3-day-old *L. sticticalis* adults demonstrated significantly increased oviposition synchronicity, and flights of varying lengths within 24 h significantly promoted reproduction [64]. After a certain period of time in flight, the ovarian development speed of immature adult female fall armyworm, *Spodoptera frugiperda* (Lepidoptera: Noctuidae), at 1–3 days was accelerated, the mating activity was advanced, and the oviposition synchronization was enhanced; flight had no obvious effect on the amount and duration of the oviposition [69]. The short flight of the rice leaf roller, *Cnaphalocrocis medinalis* (Lepidoptera: Pyralidae), was found to shorten the pre-oviposition period and increase oviposition uniformity [70]. Influenced by the occurrence time of migration flight, some insects can still carry out long-distance migration even when their ovaries are mature and they are mating. After the ovaries mature, many insects, such as *Anax junius* (Odonata: Aeshnidae) [71], *S. exigua* [63,72], and *S. frugiperda* [73], carry many mature eggs during migration flight. However, many cross-sea migratory insects trapped on BHC were mature, and the mating ratio was relatively high [48,49,50,51,52,53]. All of these data challenge the theory of oogenesis-flight syndrome.

### 3.2. Regulation of Insulin in Relation to Insect Migration and Flight

The insulin signaling pathway’s impact on migratory activities is mainly manifested in its regulation of insect wing development [18,23,74]. *N. lugens* is a major migratory agricultural pest, feeding only on rice. The adult has two wing types: the long-winged type can migrate long distances, while the short-winged type cannot fly [66]. The growth stage and nutrient content of rice plants can affect the wing type conversion of the brown planthopper, and an increased soluble sugar content in the late growth stage of rice plants can induce the generation of long-wing types of *N. lugens* and the white-backed planthopper, *Sogatella furcifera* (Homoptera: Delphacidae) [75,76,77]. The insulin signaling pathway is the main signaling pathway in insects for sensing various sugar changes.

Two insulin receptors, InR1 and InR2, exist in *N. lugens*. InR1 participates in the canonical insulin signaling process, binding to insulin-like polypeptide 3 (ILP3), which is secreted by the brain of *N. lugens*. The signal is transmitted by the phosphorylation of the phosphatidylinositol-3-OH kinase (PI3K)–protein kinase B (Akt) signaling cascade to the forkhead transcription factor Foxo in the wing buds, and the phosphorylated Foxo is trapped in the cytoplasm and does not exert biological activity. At this time, the ontogeny is long-winged. Receptor InR2 is a trans-regulatory factor that can inhibit the InR1–PI3K–Akt signaling pathway such that transcription factor Foxo enters the nucleus and activates downstream gene expression, and the ontogeny is short-winged [23]. An elevated glucose content in rice plants will stimulate increased insulin secretion in the feeding brown planthopper and eventually inhibit Foxo transcription factor activity, resulting in long-winged individuals. Conversely, when the glucose content of rice plants is low, there is less insulin secretion, and the Foxo transcription factor is in the activated state, inducing short-winged individuals [78]. The expression of genes related to the IIS–PI3K–Akt–Foxo insulin signaling pathway was significantly different between long-winged and short-winged individuals of *S. furcifera*, and related genes involved in muscle contraction and energy metabolism were highly expressed in long-winged individuals [79]. The regulation of the insulin signaling pathway in wing type development explains the phenomenon by which rice planthoppers that develop into the long-winged type escape from the undesirable habitat by migration when the nutritional status of the host plants deteriorates at the late stage of growth.

The insulin signaling pathway also plays an important role in the intra-generation wing differentiation of migratory insects. The sex-determining gene *tra-2* of female *N. lugens* adults controlled the wing type transition of offspring by regulating the insulin signaling pathway [80]. The embryonic Foxo transcription factor was found to negatively regulate the proportion of winged aphids in the offspring of the pea aphid, *Acyrthosiphon pisum* (Homoptera: Sternorrhyncha), and the insulin-like peptides ILP4, ILP5, and ILP11 showed obvious stage-specific phenomena in regulating the wing type transition of aphids across generations [81]. Further, the insulin signaling pathway can affect the adaptation of migratory insects to hypoxia. Based on a comparison of whole-genome resequencing between the plateau and plain populations of the migratory locust, *Locusta migratoria* (Orthoptera: Acrididae), it was found that the *PTPN1* gene was mutated in the plateau population and showed a positive selection effect. *PTPN1* encodes the protein tyrosine–protein phosphatase non-receptor type 1, which inhibits the biochemical process of the insulin pathway by dephosphorylation of the insulin receptor; thus, *L. migratoria* individuals maintain relatively stable insulin pathway activity and normal glucose metabolism levels and activity and thus adapt to hypoxia at high altitudes [82].

### 3.3. Regulation of the Juvenile Hormone on Insect Migration and Reproduction

For migratory insects, JH plays a key role in the regulation of migration due to the interaction between flight and reproduction [83,84]. The topical treatment of migratory insects with JH analogs can allow us to observe changes in their flight and reproductive activities and analyze specific regulatory effects. When the JH analog methoprene was applied to the body surface of 1-day-old *M. separata*, the pre-oviposition period of female moths was shortened, their reproductive ability was significantly increased, and their flight capacity was significantly inhibited [21,85]; in addition, the content of glycerides as flight energy material was significantly decreased [86]. When *M. separata* experience starvation or flight at the age of 1 day, the JH titer increases, directly promoting an increase in the oviposition and a shortened pre-oviposition period in adult moths [87]. Compared with short-time flight and no-flight treatment, long-time flight was shown to stimulate the secretion of more JH in the corpus allatum of the grasshopper, *Melanoplus sanguinipes* (Orthoptera: Acrididae), and the increased titer promoted the reproductive activity of the locust [88]. In general, a low JH content can promote the occurrence of migratory behavior, but when the titer rises to a certain threshold, JH can cause the termination of migratory flight, promote ovarian development, and enter the reproductive process of migratory insects [16,57,89]. The relative expression level of the vitellogenin gene *MsVg* in *M. separata* across-sea migrants showed significant seasonal changes and was significantly correlated with the degree of ovarian development [90]. However, the JH titer, which played a regulatory role, was negatively correlated with the degree of ovarian development. This is contrary to the theory that higher JH titers will promote the reproductive process. This discrepancy may be due to the distance between the source area and the migrant trapping site, whether migrants experienced continuous migration when trapped, and whether migrants had greater reproductive demand [90]. This also reflects the complexity of the influence of JH on the migration process.

JH regulates migration by promoting the reproductive activities of migrating insects. The core link is that JH induces the biosynthesis of vitellogenin in the fat body and promotes the receptor-mediated vitellogenin endosis of oocytes, thus promoting the maturation process of oocytes [91,92]. The accumulation of vitellogenin is the premise of oocyte maturation and the basic guarantee of embryonic development. Vitellogenin is transcribed and translated by the insect fat body in large quantities, transported by the hemolymph, and then selectively absorbed by vitellogenin receptor (VgR)-mediated endocytosis on the outer surface of developing oocytes [93,94]. The regulation of JH in the reproduction of *L. migratoria*, as a typical migratory insect, has been well studied. JH in *L. migratoria* triggers the phosphorylation of the cell polarity protein Par3 through the G-protein-coupled receptor (GPCR)–small G protein cell division cycle protein 42 (Cdc42)–atypical protein kinase C (aPKC) signaling pathway, and the phosphorylated Par3 dissociates from the core protein β-Cat of the adhesive, causing the depolymerization of zonular adherens at the junction of three cells, forming intercellular channels for Vg transport [95]. Moreover, JH in *L. migratoria* induced the phosphorylation of VgR at serine 1361 through the GPCR-coupled receptor phospholipase C-protein kinase C iota signaling pathway and then promoted the binding of phosphorylated VgR to vitellogenin, thereby affecting endocytosis [96].

In migratory insects, JH regulates the expression of related downstream genes by binding to the nuclear receptor Met and then regulates vitellogenesis and oocyte maturation. JH initiates downstream gene expression through the Met/Kr-h1 signaling pathway, which promotes fat body and ovarian development, thus regulating vitellogenesis and oocyte maturation in *L. migratoria* [97,98,99]. Met directly regulates the transcriptional expression of the cell cycle genes *Mcm4*, *Mcm7*, *Cdc6*, *Cdk6*, and *E2f1* [100,101,102]. Met also regulates the dephosphorylation of Foxo through the leucine carboxymethyltransferase 1-protein phosphatase 2A-Foxo transcription factor signal transduction pathway and then regulates the transcriptional expression of the cell cycle genes *Cdc2* and *Orc5* [103]. In *L. migratoria*, two glucose-regulated protein 78 kDa genes (*Grp78*) exist. *Grp78-1* expression is regulated by the JH–Met–Mcm4/7 pathway, while *Grp78-2* expression is directly activated by the JH receptor complex [104]. JH affects the polyploidization of locust fat body cells by regulating the transcriptional expression of these genes and then regulates the massive biosynthesis of vitellogenin, promoting vitellogenesis and oocyte development and maturation.

## 4. Energy and Substance Metabolism during Insect Migration Flight

**Figure 2:**
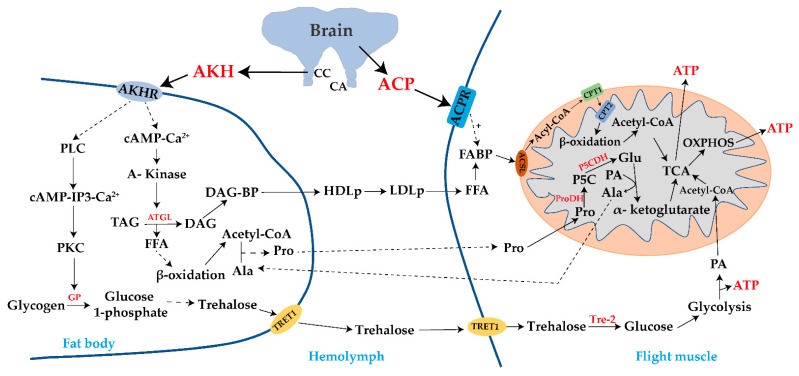
Metabolic regulation process of energy substances during insect migration flight. ACP: AKH/corazonin-related neuropeptide; ACPR: ACP receptor; ACSL: acyl-CoA synthetase; ATP: adenosine triphosphate; CPT1: carnitine palmitoyltransferase 1; CPT2: carnitine palmitoyltransferase 2; AKH: adipokinetic hormone; AKHR: AKH receptor; Ala: alanine; CA: corpus allatum; cAMP: cyclic adenosine monophosphate; CC: corpora cardiaca; DAG: diacylglycerol; DAG-BP: DAG binding protein; FABP: fatty acid binding protein; FFA: free fatty acids; Glu: glutamate; GP: glycogen phosphorylase; HDLp: high-density lipoprotein; IP3: inositol-1,4,5-trisphosphate; LDLp: low-density lipoprotein; OXPHOS: oxidative phosphorylation; PA: Pyruvic acid; PKC: protein kinase C; PLC: phospholipase C; Pro: proline; ProDH: proline dehydrogenase; P5C: pyrroline-5-carboxylate; P5CDH: pyrroline-5-carboxylate dehydrogenase; TAG: triacylglycerol; TCA: tricarboxylic acid cycle, Tre-2: trehalase-2; TRET1: trehalose transporter 1.

Insects need to consume a lot of energy during flight, when the metabolic rate is much higher than it is at rest [10]. The flight muscle is the main region of energy expenditure during flight, and energy substances are transported from the fat body through the hemolymph to the flight muscle cell [105]. The continuous and stable metabolic regulation of the energy supply is the basis by which many migratory insects achieve long-distance flight. The process of the metabolic regulation of energy substances during insect migration flight is shown in Figure 2.

### 4.1. Energy Substances during Insect Flight

The main energy substances of insects include carbohydrates, lipids, and amino acids [10,106,107]. Most insects, including Diptera, Hymenoptera, and some Lepidoptera, mainly use carbohydrates to provide energy for short-distance flight. Many long-distance migratory insects, including *L. migratoria* [108], *M. sanguinipes* [109], *A. ipsilon* [84,110], *D. plexippus* [111], *M. separata* [112], *S. exigua* [113], *S. litura* [114], and *S. exempta* [115], are generally powered by carbohydrates at the initial stage of flight activities; then, they mainly use the lipid substances stored in the fat body as the energy supply for continuous long-distance flight [10,116,117,118]. Some insects, such as the Colorado potato beetle, *Leptinotarsa decemlineata* (Coleoptera: Chrysomelidae) [119,120], and some bees and wasps [121,122], can also use proline oxidative metabolism to supply energy for flight.

### 4.2. Amino Acid Energy Metabolism during Flight

Potato beetle adults can fly long distances to cause harm in different planting areas [123], and their long-distance flight is powered by proline [119,120]. Proline in flight muscle is oxidized to glutamate by active proline dehydrogenase. Under the action of alanine aminotransferase, the amino acid of glutamate is transferred to the transamination of pyruvic acid (PA), producing alanine and α-ketoglutarate, which then enter the tricarboxylic acid cycle (TCA) and oxidize for energy supply [122,124]. The oxidation of proline in the flight muscle produces alanine, which is rapidly circulated to the fat body and bonded to acetyl CoA produced by the β oxidation of lipids and propagates and generates proline, with high solubility in the hemolymph, which is transported to the flight muscle for repeated proline metabolism for energy supply [122,124].

### 4.3. Carbohydrate Metabolism during Flight

Glycogen and trehalose are the main carbohydrate reserves in insects and are mainly stored in the hemolymph, fat body, and intestine, with a small amount present in the muscle [124]. When insects use carbohydrates as the energy supply during flight, glycogen stored in the fat body and other tissues can be rapidly converted into trehalose, which enters the hemolymph and is further transported to flight muscle, where it is converted into glucose for energy [124,125]. Trehalose synthesis in insects is mainly achieved through the trehalose-6-phosphate synthase/trehalose-6-phosphate phosphatase (TPS/TPP) pathway [126,127]. In the insect fat body, glycogen is hydrolyzed by glycogen phosphorylase (GP) to produce glucose 1-phosphate. Glucose 1-phosphate and uridine triphosphate are catalyzed by uridine diphosphate glucose–glucose pyrophosphorylase to synthesize uridine diphosphate glucose. Uridine diphosphate glucose and glucose 6-phosphate are catalyzed by trehalose 6-phosphate synthase to produce trehalose 6-phosphate and release uridine diphosphate. Subsequently, trehalose 6-phosphate phosphatase catalyzes the dephosphorylation of trehalose 6-phosphate to form trehalose and phosphate [124,126,127].

Trehalose transporter 1 (Tret1) can perform transmembrane transport in response to intracellular and extracellular trehalose concentration gradients, thus performing a specific function [128]. In *N. lugens*, the trehalose transporter NlTret1 plays an important role in trehalose-specific transport and energy supply [129,130]. During insect flight, trehalose is transported to the flight muscle and then decomposed into glucose by trehalase [126,131]. Trehalase is the only enzyme that catalyzes the trehalose decomposition of trehalose and plays an important role in regulating metabolism. There are two types of trehalase: free soluble trehalase Tre-1 and membrane-bound trehalase Tre-2 [132,133]. Trehalase is distributed in the midgut, hemolymph, ovary, flight muscle, and other tissues of insects and has different spatiotemporal expression dynamics [126,133,134]. The main function of intracellular soluble Tre-1 is to decompose trehalose in cells, whereas membrane-bound Tre-2 is a transmembrane protein whose main function is to degrade extracellular trehalose and provide energy for midgut and muscle movement [126]. Glucose undergoes glycolysis to produce PA and a small amount of ATP, and PA then enters the mitochondria and is oxidized by TCA to produce a large amount of energy to support insect flight [107,124].

### 4.4. Lipid Compound Metabolism during Flight

Lipids are the most economical energy reserve for long-distance and continuous insect flight [105,107,116]. The metabolic regulation process of lipid substances during *L. migratoria* flight has been well studied [14,135]. Glycogen and glucose levels in the flight muscles of *L. migratoria* gradually decrease to a constant level during the first 15 min of continuous flight. After 30 min of continuous flight, lipids gradually accumulate in the hemolymph, and high-calorie lipids begin to serve as the main energy supply substances [14,136]. This also suggests that when the flight energy supply changes from carbohydrates to lipids, sugars are not simply consumed, but a certain level of basic metabolism is maintained.

Triacylglycerol (TAG) makes up about 90% of the lipids stored in the insect fat body. During flight, the catalysis of TAG by adipose triglyceride lipase (ATGL) to produce diacylglycerol (DAG) is the main form of lipid transport [137]. DAG binds to apolipoproteins to form lipoproteins, which are transported to the flight muscle through the hemolymph [11,124,138]. As a means of lipid transport, apolipoproteins can be divided into carrier protein I (apoLp-I), carrier protein II (apoLp-II), and carrier protein III (apoLp-III) according to the particle size and density [124,139,140]. Diacylglycerol is assembled with apoLp-I and apoLp-II to form high-density lipoprotein (HDLp), which is released into the hemolymph. High-density lipoprotein combines with apoLp-III to convert high-density lipoprotein into low-density lipoprotein (LDLp) and release DAG into the flight muscle. Then, apoLp-III dissociates from the complex and participates in new DAG transport with high-density lipoprotein [124,135,141].

Membrane-bound esterase on flight muscle cells can bind to lipoproteins and catalyze the decomposition of DAG into free fatty acids (FFA) and glycerol [124,135,142,143], and FFA are transported between the cytoplasm and mitochondria by binding to the fatty acid binding protein (FABP) in the cytoplasm [144,145,146]. Insect flight can induce a significant increase in the transcription and expression of the *FABP* gene [147]. In both desert locust *Schistocerca gregaria* (Orthoptera: Acrididae) and *L. migratoria*, the flight duration of individuals was significantly shortened after *FABP* gene expression was silenced. The flight distance of *L. migratoria* was also significantly decreased [14,148], mainly due to the silencing of the *FABP* gene, which seriously affects the oxidative metabolism of fatty acids in the flight muscle during sustained flight. FFA are catalyzed by fatty acyl-coenzyme A on the outer mitochondrial membrane to produce fatty acyl A, which is then transported to the mitochondrial matrix by carnitine palmitoyltransferase (CPT) in the inner and outer membranes of mitochondria [136,149,150]. Then, acetyl CoA is generated by β oxidation and is oxidized for energy through the TCA cycle [14,124,151,152].

### 4.5. Hormonal Regulation of Energy Substances during Flight

The metabolism of energy substances in insects is regulated by a variety of neuropeptide hormones such as insulin-like hormones, AKH, AKH/corazonin-related neuropeptide (ACP), and octopamine (OA) [12,14,107,124,153]. Among them, insect insulin-like hormones can promote the absorption of glucose and the conversion of glucose into glycogen, inhibit gluconeogenesis, promote the synthesis reaction of proteins and fatty acids, and inhibit the decomposition reaction [13,154]. In contrast, OA, AKH, and ACP regulate the metabolic energy supply process of carbohydrates and lipids and directly affect the flight process [14,107]. Insect flight is a process of high energy consumption, which requires the rapid and efficient mobilization, transfer, and utilization of energy storage substances. Therefore, the process of regulating neuropeptide hormones such as OA, AKH, and ACP in the metabolism of the flight energy substances of migratory insects is discussed here.

OA is a weakly basic low-molecular-weight nitrogen-containing compound. Tyrosine is decarboxylated by tyrosine decarboxylase to produce tyramine, which is further decarboxylated by tyrosine β hydroxylase, forming OA [155,156]. As a neurotransmitter, neuromodulator, and neurohormone, OA is widely distributed in insects, especially in various ganglia and neurons of the central nervous system [157]. It regulates the level of the intracellular second messenger cAMP or Ca^2+^ through GPCR and affects various behaviors and physiological processes of insects, such as courtship, flight, oviposition, learning, metabolism, and immune response [156]. In migratory insects, OA can be involved in regulating the energy metabolism of flight muscles and the fat body, thus affecting flight movement processes [156,158,159].

The glycolysis of carbohydrates supplies energy for the initial flight activities of long-distance migratory insects. Phosphofructokinase is a key enzyme in the glycolysis process. The combined action of adenosine monophosphate (AMP) and fructose 2,6-diphosphate can activate phosphofructokinase to stimulate the glycolysis process [124,160,161]. Within the first few minutes of flight, the concentration of fructose-2,6-diphosphate in the flying muscle of *L. migratoria* was found to decrease sharply, resulting in decreased phosphofructokinase activity [160,162], while the exogenous injection of OA into the hemolymph led to a rapid increase in fructose-2,6-diphosphate in the flying muscle, counteracting its consumption during flying. However, high doses of OA and flight activity can lead to decreased fructose 2,6-diphosphate content [162]. The central dorsal unpaired median (DUM) neurons are the main OA release neurons acting on the flight muscle. The inhibition of DUM neurons activity reduces the level of OA in the flight muscle, and, thus, glycolytic activity activated by OA is reduced [163,164,165]. The release of OA by DUM neurons regulates glycolysis only during the take-off phase of insect flight [14,166]. Therefore, the inhibition of continuous flight by the activity of DUM neurons causes a decrease in fructose 2,6-diphosphate, and the decreased glycolytic activity will cause the conversion of energy supply substances from carbohydrates to lipids during the sustained flight of long-distance migratory insects. In addition, OA can participate in regulating the metabolic process of the fat body and affect flight activities, which is mainly reflected in stimulating the secretion and release of AKH from the corpora cardiaca. In addition, OA can directly act on the fat body and induce the release of fatty acids into the hemolymph. Relevant studies have been reviewed previously [14,156,163]. During sustained flight, the initial increase in hemolymph lipids is caused by a temporary increase in OA, but the increase in lipids in the hemolymph after sustained flight is caused by the release of AKH [167,168].

AKH is a class of neuropeptides mainly synthesized, stored, and released into the hemolymph by the corpora cardiaca located at the back of the insect brain. Energy consumption during flight will stimulate the secretion of AKH, glucagon-like peptide. AKH needs to bind to receptor proteins on the cell membrane (AKHR) to play its role. AKHR is composed of seven transmembrane α-helices and belongs to the GPCR family [169,170]. After the action of AKH on AKHR, GP is activated by intracellular second messenger transduction, which catalyzes the hydrolysis of glycogen to produce glucose 1-phosphate and finally converts it to trehalose, which provides energy for insect flight [171]. Induced by flight activity, AKH is largely released into the hemolymph within 10 min after the initiation of *L. migratoria* flight, and glycogen in the fat body is hydrolyzed by AKHII to produce trehalose, which is supplemented into the hemolymph [172]. After silencing the expression of AKHR in *N. lugens*, the level of DAG in the hemolymph decreased, and the level of TAG in the fat body increased, which shows the important role of AKHR in the lipid metabolism in the brown planthopper [173]. The extract of *L. migratoria* can significantly increase the lipid content in the hemolymph [174], and AKHRI, AKHRII, and AKHRIII have been isolated and identified from the extract of *L. migratoria* [175,176,177]. There are also three types of AKH in desert locusts. After a long flight, the *AKH* gene expression and AKH content in the hemolymph of *L. migratoria* and *S. gregaria* increased correspondingly [177,178]. After the activation of AKHR and AKH, the intracellular Ca^2+^ level and the concentration of cAMP increase, followed by signaling via the cAMP-dependent protein kinase (PKA) pathway, or the phospholipase C-inositol-1,4,5-trisphosphate/Ca^2+^-protein kinase C (PKC) pathway activates PKC, which in turn activates GP and TAG esterase [171,179,180,181,182], initiating carbohydrates and lipids metabolic processes and promoting glycogen and triglycerides hydrolysis to provide energy for flight. AKHII and AKHI induce the hydrolysis of glycogen and TAG, respectively, to provide energy for the continuous flight of *L. migratoria* [136,183,184]. In tobacco armyworm *Manduca sexta* (Lepidoptera: Sphingidae), AKH regulated the activation of GP in starved larvae and lipid mobilization during flight in adults [185]. The injection of synthetic AKH into male *V. cardui* caused an increase in the lipid concentration but no significant change in the total carbohydrate content in the hemolymph [186]. Therefore, AKH mainly regulates and affects the flight of long-distance migratory insects by mobilizing lipid energy substances in the fat body.

ACP was named because of its high structural similarity to the neuropeptides AKH and corazonin [187]. ACP is synthesized by anterior brain neurons, transported and stored in the corpora cardiaca, and finally released into the hemolymph [14,187]. The signaling system of ACP and its receptor protein ACPR is not widely found in insects; this was reported only for some insects in the orders Diptera, Lepidoptera, Orthoptera, Coleoptera, Hymenoptera, and Hemiptera [187]. The biological function of ACP in *L. migratoria* has been well studied [153,188]. Siegert isolated a new neuropeptide from the corpora cardiaca of *L. migratoria* and injected the neuropeptide into adult locusts, which did not cause changes in the trehalose levels in the hemolymph. However, the injection of the neuropeptide into cockroaches led to increased trehalose levels in the hemolymph, so the neuropeptide was named locust hyperglycemic hormone (LomHrTh) at that time [188]. Hou et al. proved that the site of ACP neuropeptide synthesis in *L. migratoria* is the neurosecretory cells in the forebrain region, rather than the corpora cardiaca. In addition, the sustained flight capability of *ACP* and *ACPR* mutant locusts was compared, and the expression and metabolite levels of lipid-metabolism-related genes were measured. It was revealed that the ACP polypeptide combined with the ACPR of the flight muscle affected the function of FABP and regulated the lipid transport and oxidation process, thus regulating the long-distance sustained flight of locusts [153]. ACP mainly promotes the oxidative metabolism of fatty acids in flight muscle cells and affects the continuous flight activity of locusts. However, whether the regulatory mechanism of the ACP peptide in the lipid metabolism is conserved among migratory insects and whether it also affects the long-distance flight process have yet to be investigated.

## 5. Prospect

Insects can escape from undesirable habitats by migrating, which is a countermeasure by which they can adapt to resource and environmental changes over long periods of time. In addition, with high reproductive ability, insects often experience rapid population growth after migration, and the concentrated migration of agricultural pests can cause outbreaks and agricultural damages. The migration of natural enemies and pollinators has a great impact on the inter-regional material exchange, energy transport, and stability of regional ecosystems. Insect migration is a complex ecological phenomenon, often characterized in terms of sudden increases and sudden decreases. The migration of some major agricultural pests involves a wide regional scale, large populations, and a large amount of harm, and it is difficult to monitor and predict their populations. At present, our understanding of insect migration processes, especially air flight behavior, the change law of the large-scale migration population structure, and the physiological basis and regulation mechanism of insect behavior, is still insufficient, and these aspects need to be further explored.

Insect species are extensive and diverse, and the ecosystems of study areas around the world are complex and diverse. The migration of insects is closely related to the specific habitat environment, the high-altitude meteorological conditions of migratory flight, and the environmental factors of the landing site, among other factors [3]. Therefore, determining how to dynamically monitor the population structure and the number of migrating insects in real time and how to combine the dynamic changes in insect populations and their interaction with environmental factors to accurately predict the migration process has important practical significance for guidance in production. At this stage, the real-time monitoring of large-scale insect migration can be realized using tools such as a weather radar and a high-precision insect-specific radar [189]. In addition, long-term monitoring at multiple sites and across regions would provide a basis for understanding the change process and laws of insect migration [190].

Omics studies have shown that genetic information related to energy metabolism, the interaction between migration and reproduction, hormone synthesis, etc. is the molecular basis for the long-distance migration of insects [83,84,191,192,193]. However, the key environmental factors that induce insect migration are still unclear. The process of migrating insects perceiving changes in the environment and the regulatory mechanism of endocrine hormones in their flight and reproductive system also need to be further studied.
